# Post-Dialysis Fatigue Is Not Associated with Serum Lactate Levels in Patients on Chronic Hemodialysis

**DOI:** 10.3390/jcm14082706

**Published:** 2025-04-15

**Authors:** Maurizio Bossola, Nunzia Ciferri, Ilaria Mariani, Tania Monteburini, Stefano Santarelli, Enrico Di Stasio

**Affiliations:** 1 Servizio Emodialisi, Dipartimento di Scienze Mediche e Chirurgiche, Università Cattolica del Sacro Cuore, 00168 Roma, Italy; 2 Policlinico Universitario Fondazione Agostino Gemelli IRCCS, 00168 Roma, Italy; nunziagiferri@gmail.com (N.C.); ilaria.mariani04@icatt.it (I.M.);; 3 Divisione di Nefrologia, Servizio Emodialisi, Dipartimento di Scienze Mediche e Chirurgiche, Università Cattolica del Sacro Cuore, 00168 Roma, Italy; 4 Dipartimento di Nefrologia, Ospedale “Carlo Urbani” Jesi, 60035 Jesi, Italy; 5 Dipartimento di Scienze Biotecnologiche di Base, Cliniche Intensivologiche e Perioperatorie, Università Cattolica del Sacro Cuore, 00168 Roma, Italy

**Keywords:** hemodialysis, fatigue, post-dialysis fatigue, lactate, acidosis

## Abstract

**Background/Objectives:** To measure the peri-dialytic serum lactate, sodium, potassium, calcium, and pH and base excess in chronic hemodialysis patients with and without post-dialysis fatigue (PDF). **Methods**: Patients were asked “Do you feel fatigued after dialysis?” Each patient was invited to rate the intensity, duration, and frequency of PDF from one to five. The recovery time after the hemodialysis session (TIRD) was calculated, and inviting patients were to answer the following single open-ended question: “How long does it take you to recover from a dialysis session?” Pre- and post-dialysis arterial blood was sampled, and pH, bicarbonates, base excess, sodium, calcium, potassium, and lactate were measured. **Results:** One hundred fifty-eight patients were included in the study. One hundred seventeen patients declared to suffer from PDF and forty-one did not. Median [range] PDF frequency, intensity, duration, and TIRD were 5 (1–5), 4 (1–5), 3 (1–5), and 12 h (1–48), respectively. Seventy patients had a TIRD ≤ 12 h and forty-seven had a TIRD > 12 h. Median post-dialysis and post-dialysis/pre-dialysis difference serum lactate levels (mmol/L) did not differ between patients with and without PDF (*p* = 0.111 and *p* = 0.395, respectively). In addition, the distribution of patients according to post-dialysis serum lactate levels was similar in the presence or absence of PDF. The median post-dialysis and post-dialysis/pre-dialysis difference serum lactate concentrations did not differ significantly according to the score of the PDF intensity and PDF duration (*p* = 0.928 and 0.935, *p* = 0.610 and 0.548, respectively). Finally, we stratified patients into two groups according to the length of TIRD: ≤12 h and >12 h. The median post-dialysis serum lactate concentrations did not differ significantly between the two groups (*p* = 0.862) as well as the median post-dialysis/pre-dialysis difference (*p* = 0.583). Also, the distribution of patients according to post-dialysis serum lactate levels was similar in the two groups. **Conclusions:** PDF and TIRD are not associated with peri-dialytic changes in serum lactate in patients on chronic hemodialysis.

## 1. Introduction

Many patients, following a hemodialysis treatment, report to feel tired and the need for a rest or sleeping time. This condition, conventionally called post-dialysis fatigue (PDF), has been described as a feeling of being worn out, drained, or exhausted [1,2,3]. PDF is one of the most debilitating symptoms of hemodialysis patients, significantly impairing their quality of life and causing frustration and depression [1,2,3].

PDF is common, although prevalence estimates vary between studies from 20% to 86%, and differences in estimates are likely related to different inclusion criteria, ascertainment methods, or definitions of fatigue [1,2,3].

The causes and the pathogenesis of PDF are essentially unknown [1,2,3,4]. However, some mechanisms have been proposed, such as (1) the rapid decline in osmolarity occurring during dialysis as a result of the combined effect of a reduction in serum urea and sodium concentration (this leads to brain swelling and headache, restlessness, nausea, muscle cramps, and fatigue) [5,6]; (2) the release of cytokines such as interleukin-1, interleukin-6, and tumour necrosis factor-alpha [7,8,9]; and (3) the accumulation of metabolites and toxins in the muscle. In addition, the data point also to psychological factors contributing to PDF [1,2,3,4]. Referring to the third point, it has been suggested that intradialytic hypotension, one of the most common complications of hemodialysis, may contribute to muscle ischemia, accumulation of lactate in the muscle, high serum lactate levels, and consequent muscle fatigue and PDF [10,11,12,13]. These data are supported by the observation that higher levels of post-dialysis lactate are associated with PDF in patients on chronic hemodialysis and, in particular, that post-dialysis lactate levels in patients with severe PDF were significantly higher than that in patients with mild PDF [12]. It is hypothesised that microcirculatory dysfunction can cause regional tissue hypoxia and consequent hyperlactatemia [12]. Unfortunately, to the best of our knowledge, there is no evidence of other studies on this issue. The present study aims to measure the peri-dialytic serum lactate concentrations in end-stage renal disease (ESRD) patients on chronic hemodialysis and to define the possible correlation with PDF prevalence and characteristics as well with the length of the time of recovery after dialysis (TIRD) defined as the time taken to recover from a dialysis session.

## 2. Patients and Methods

All prevalent ESRD patients receiving chronic hemodialysis at our hospital in December 2024 were eligible for inclusion in the study. Exclusion criteria were as follows: dialysis duration <1 year, diagnosis of dementia based on DSM-IV criteria, presence of acute infectious disease, presence of active cancer, vascular access though a central venous catheter, heart failure, respiratory failure, haemorrhage, severe infections, alcohol abuse, shock, and liver disease [13]. The study was performed in adherence to the Declaration of Helsinki, and the protocol was approved by the local ethics committee (Prot ID 3169). Written informed consent was obtained from all participants before enrolment in the study. For each participant, the following parameters were recorded at the time of inclusion in the study: age, gender, underlying renal disease, weight, height, hemodialysis regimen, type and number of comorbid conditions, and the Charlson Comorbidity Index [14].

### 2.1. Identification and Grading of PDF

The assessment of PDF was conducted according to the studies of Sklar et al. [15]. Each patient was interviewed during one of the patient’s regularly scheduled treatments. Patients were suffering from PDF if they spontaneously offered this complaint when asked the open-ended question: “Do you feel fatigued after dialysis?” Then, each patient was invited to rate the intensity, duration, and frequency of PDF from 1 to 5.

### 2.2. Other Measurements

The recovery time after the hemodialysis session (TIRD) was calculated according to Lindsay et al. [16]. Briefly, patients were invited to answer to the following single open-ended question: “How long does it take you to recover from a dialysis session?”

Functional ability was estimated using the Katz activities of daily living (ADLs), and the Lawton and Brody scale for instrumental activities of daily living (IADLs) [17,18]. These scales are most adopted for assessing functional independency for clinical and epidemiological purposes; disability in the ADLs was defined as need of assistance for performing two or more ADLs. The reason for not choosing a single-point decline is that impairment in two ADLs is less likely to capture physiological fluctuations in functional performance. Impairment in IADL function was identified by a score <7; this higher cut-off level is generally adopted to avoid a “floor effect”. The ADL scale is based on seven levels of self-performance including dressing, eating, toilet use, bathing, mobility in bed, locomotion, and transfer. Similarly, the IADL scale is based on seven levels of self-performance including meal preparation, housework, managing finance, phone use, shopping, transportation, and managing medications. Finally, the Kt/V was calculated according to a standard formula and used as an index of adequacy of the hemodialysis treatment.

### 2.3. Hemodialysis

All patients received conventional 4 h bicarbonate hemodialysis three times a week. The blood flow ranged from 250 to 300 mL/min with a dialysis rate flow of 500 mL/min. All patients were treated with high-permeability membranes.

### 2.4. Laboratory Measurements

Blood samples were obtained from HD patients directly through the arteriovenous fistula before their scheduled HD session at the beginning of the week. Serum was separated within 30 min, and samples were kept frozen at −70 °C if not analysed immediately. Laboratory parameters were measured by routine methods at the Department of Diagnostic and Laboratory Medicine, Unity of Chemistry, Biochemistry and Clinical Molecular Biology. Pre- and post-dialysis arterial blood was sampled from the arterial blood line of the arteriovenous fistula, and the following parameters were tested: pH, actual bicarbonate, base excess, sodium, potassium, and lactate (arterial blood gas analysis).

### 2.5. Statistical Analyses

Statistical analysis was performed using the Statistical Package for Social Science (SPSS), release 15.0. Continuous variables were expressed as mean ± SD, categorical variables displayed as frequencies, and the appropriate parametric or non-parametric test was used to assess significance of the differences between groups. All data were first analysed for normality of distribution using the Kolmogorov–Smirnov test of normality. Correlations were calculated with the Spearman’s rho correlation coefficient. After adjustment for multiple measures, a *p*-value < 0.01 was considered statistically significant. Correlation Matrix of Fatigue and hemogas analysis parameters were built, reporting Spearman’s rho and significance values. The primary outcome was defined as the difference between pre- and post-dialysis serum lactate levels according to the presence of PDF or TIRD class (≤ or >12 h). Considering α = 0.05, power = 80%, and the number of enrolled subjects, effect sizes of 0.51 and 0.53 were calculated for PDF presence and TIRD class, respectively; our observed effect sizes (0.1 units for both variables) are significantly lower, thus confirming the absence of association between lactate and PDF or TIRD. As shown in Table 1, no differences in baseline demographical, clinical, and laboratory characteristics between PDF presence and absence subgoups were detected, thus warranting no further confounding factor controls through the data analysis.

## 3. Results

One hundred fifty-eight patients were included in the study. Their demographic, clinical, and laboratory characteristics are shown in Table 1. One hundred seventeen patients declared to suffer PDF (group A) and forty-one did not (group B). Median [95% CI] PDF frequency was four (five to five). Median [95% CI] PDF intensity was three (three to four). Median [95% CI] PDF duration was three (two to three). The median [95% CI] TIRD was 12 h (11.9 to 12). Seventy patients had a TIRD ≤ 12 h and forty-seven had one >12 h.

The pre-dialysis and post-dialysis serum pH and concentrations of bicarbonates, sodium, calcium, potassium, lactate, and base excess are shown in Table 2, either in patients with or without PDF. As expected, in both groups (A and B), a significant increase in pH, bicarbonates, calcium, sodium, and base excess and a significant decrease in potassium and lactate was observed between pre- and post-dialysis assessments.

As shown in Table 3, the median [95% CI] post-dialysis serum lactate levels (mmol/L) and the median [95% CI] Δ post-dialysis/pre-dialysis serum lactate levels (mmol/L) did not differ between patients with and without PDF. In addition, the distribution of patients according to post-dialysis serum lactate levels was similar in the presence or absence of PDF (Table 4).

The median [95% CI] post-dialysis serum lactate concentrations did not differ significantly according to the score of the PDF intensity and PDF duration (Table 5). In addition, the median [min–max] Δ post-dialysis/pre-dialysis serum lactate levels (mmol/L) were similar with regards to the PDF characteristics (Table 6).

**Figure 1:**
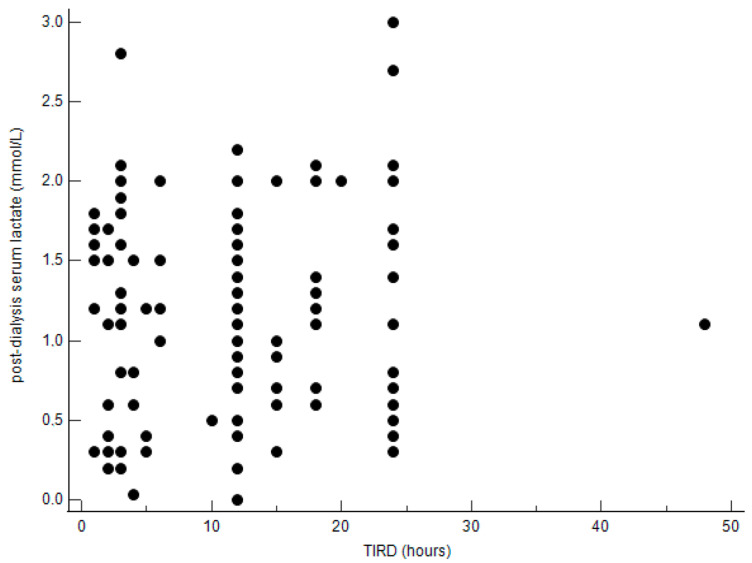
Correlation between TIRD and post-dialysis serum lactate levels. Correlation coefficient = 0.02504; *p* = 0.790.

**Figure 2:**
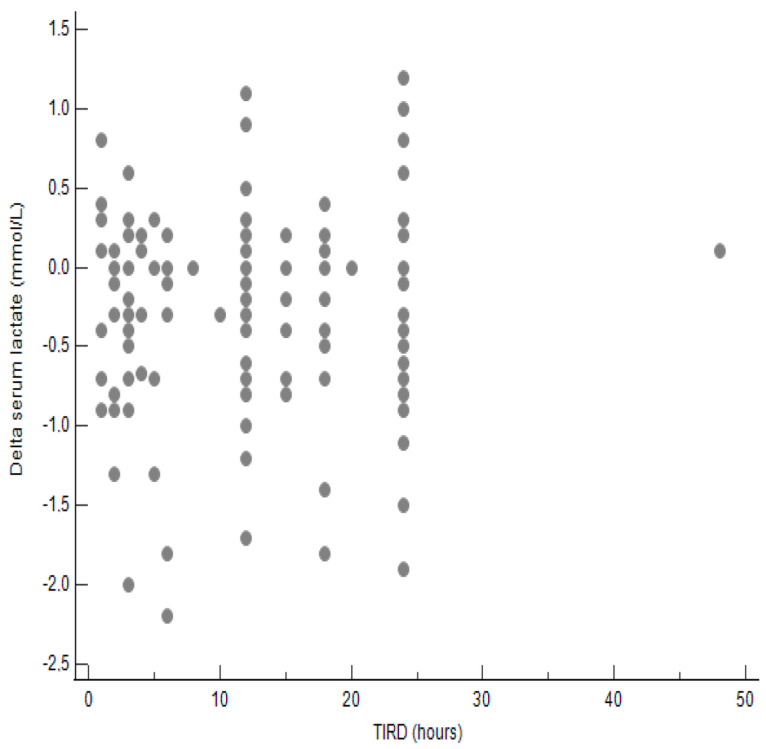
Correlation between TIRD and Δ post-dialysis/pre-dialysis serum lactate levels. Correlation coefficient = 0.017; *p* = 0.843.

Then, we stratified patients into two groups according to the length of TIRD: ≤12 h and >12 h. The median [95% CI] post-dialysis serum lactate concentrations did not differ significantly between the two groups as well as the median [min–max] Δ post-dialysis/pre-dialysis serum lactate levels (mmol/L) (Table 7). Also, the distribution of patients according to post-dialysis serum lactate levels was similar in the two groups (Table 8). As shown in Figure 1 and Figure 2, the correlations between TIRD and post-dialysis serum lactate levels and between TIRD and Δ post-dialysis/pre-dialysis serum lactate levels, respectively, were not statistically significant.

Any significant associations between post-dialysis pH, serum base excess, and serum sodium and serum potassium levels with PDF, PDF characteristics, and TIRD were observed.

## 4. Discussion

The present study shows that the difference in peri-dialytic levels of serum lactate as well as the post-dialysis serum lactate levels are not associated with PDF frequency, PDF characteristics, and TIRD in patients on maintenance hemodialysis. These results are inconsistent with those of Zu et al., showing that mean post-dialysis serum lactate levels were significantly higher in patients with severe PDF than in those with no or mild PDF, and that higher post-dialysis lactate levels were associated with PDF [12]. With respect to the study of Zu et al., we did not stratify patients according to the severity of PDF (mild or severe). Alternatively, according to the study of Sklar et al. [3], we invited patients to grade the intensity, duration, and frequency of PDF on a scale from one to five. Interestingly, the median [95% CI] post-dialysis serum lactate concentrations did not differ significantly according to the score of the PDF intensity and PDF duration, and the the median [min–max] Δ post-dialysis/pre-dialysis serum lactate levels (mmol/L) were similar with regards to the PDF characteristics. This methodological difference in the assessment of PDF severity may, at least in part, explain the different results between our study and the study of Zu et al. [12].

Reference limits for serum lactate are reported as >2 mmol/l [19]. The present study demonstrates that the frequency of post-dialysis serum lactate >2 mmol/l and >4 mmol/L is similar in patients with and without PDF and in patients with TIRD length below and above the median value. Similarly, in the study of Zu et al., the frequency of post-dialysis serum lactate levels >2 mmol/l did not differ significantly among patients with no PDF, mild PDF, and severe PDF [12].

The results of the present study argue against the hypothesis that PDF may be the results of peripheral muscle metabolic alterations related to serum lactate levels. In fact, it has been suggested that intradialytic hypotension, one of the most common complications of hemodialysis, may contribute to skeletal muscle ischemia and consequent lactate accumulation, high serum lactate levels, muscle fatigue, and PDF [1,2,3,20,21]. In the present study, hypotensive event frequency did not differ significantly between patients with and without PDF, and this might explain, at least in part, the lack of association between PDF and serum lactate levels. Generally, accumulation of lactate in skeletal muscle and the consequent decrease in cellular pH have been considered to contribute to muscle fatigue [20,21,22]. However, the contemporary view is that lactate is no longer considered a metabolic waste product and cause of muscle fatigue but instead a myokine or exerkine with autocrine-, paracrine-, and endocrine-like functions [23,24,25,26].

In the present study, we also show that the association between the differences in peri-dialytic serum sodium concentrations with PDF, PDF characteristics, and TIRD were not statistically significant. Interestingly, some authors found that the post-dialysis serum sodium levels were significantly lower in patients with PDF than in patients without [12]. This discrepancy between our study and the study of Zu et al. [12] is difficult to explain and requires further study. Nevertheless, there is evidence that low dialysate sodium concentration leading to low post-dialysis serum sodium concentration is not associated with post-dialysis fatigue or general fatigue in patients on chronic hemodialysis [27,28].

Metabolic acidosis is commonly retained to be associated with fatigue in ESRD patients on chronic hemodialysis [29]. Indeed, there is lack of evidence to support this statement. Nevertheless, the observation in the present study of any significant association between the difference in peri-dialytic pH and serum base excess with PDF, PDF characteristics, and TIRD argues against this hypothesis. In addition, the increase in in intracellular muscle acidosis has been demonstrated not to be a central factor underlying the impaired contractile function in fatigued mammalian muscle [30].

We also found significant association between the difference in peri-dialytic serum potassium and PDF, PDF characteristics, and TIRD. It has been demonstrated, recently, that the membrane in patients on chronic hemodialysis is depolarised, mainly due to hyperkalemia, and it has been argued that this potassium-induced depolarisation resembles that in fatigued muscle [31]. In the present study, the pre-dialysis serum potassium levels (mmol/L) were 4.9 (4.6–5.2) and 4.9 (4.7–5.3) in patients with and without PDF, respectively, and this may possibly explain the lack of association with PDF and TIRD.

The present study has some limitations. First, being observational and crossover, no conclusion can be drawn about causality. Second, the single center of the study population can restrict generalisability. Third, the study relied on self-reported measures for PDF and TIRD. Nevertheless, numerous studies, in the past, have shown that these measures are accurate and reliable [3,4,6,8,15,16,28,30,32,33,34,35].

In conclusion, PDF is not associated with pre-dialysis, post-dialysis, and peri-dialytic changes in serum lactate in patients on chronic hemodialysis. Post-dialysis fatigue in chronic hemodialysis patients is a complex phenomenon and a multifactorial origin, with relevant roles of behavioural, social, and psychological factors [36]. Therefore, it is amenable to consider both, biochemical and psychosocial variables, when trying to unravel the aetiology of PDF in patients on chronic hemodialysis.

## Figures and Tables

**Table 1:** Baseline demographical, clinical, and laboratory characteristics. Data are shown as mean ± standard deviation or median [95% CI for the median] for continuous variables. PDF: post-dialysis fatigue; CCI: Charlson Comorbidity Index; ADL: activity of daily living; IADL, instrumental activity of daily living.

	PDF Absent (n = 41)	PDF Present (n = 117)	*p*
Age (years)	66.2 ± 10.9	68.5 ± 11.2	0.256
Sex: male/female	31:10	73:44	0.179
Dialysis age, months	47.5 ± 45.6	54.5 ± 53.2	0.453
Primary cause of ESRD (n)			
hypertension	16	42	
glomerulonephritis	10	28	
diabetes	8	22	
interstitial nephritis	4	10	
polycystic kidney disease	2	9	
others/unknown	1	6	0.964
BMI	23.3 ± 3.3	23.8 ± 3.5	0.482
CCI	2 (0–6)	2 (0–7)	0.927
ADL	5 (0–5)	5 (0–6)	0.786
IADL	6 (0–8)	6 (0–7)	0.457
TIRD (hours)	2 (2 to 2)	12 (12 to 12)	<0.001
Weight gain (Kg)	2.7 ± 1.2	2.4 ± 1.3	0.254
Kt/Vurea	1.28 ± 0.4	1.32 ± 0.3	0.562
QB (mL/min)	268 ± 31	258 ± 34	0.146
Haemoglobin (g/dL)	10.8 ± 0.4	10.7 ± 0.6	0.377
Serum albumin (mg/dL)	3.63 ± 0.4	3.71 ± 0.5	0.412
Serum creatinine (mg/dL)	10.8 ± 2.5	11.3 ± 3.4	0.443
Dialysate sodium (mmol/L)	139 ± 1.2	139 ± 1.6	0.745
Dialysate temperature (°C)	36.4 ± 0.3	36.5 ± 0.4	0.195

**Table 2:** Laboratory variables and PDF. PDF: post-dialysis fatigue. Data are shown as median [95% CI].

	PRE-DIALYSIS			POST-DIALYSIS		
	PDF NO	PDF YES	*p*	PDF NO	PDF YES	*p*
pH	7.33 (7.31–7.46)	7.33 (7.31–7.34)	0.288	7.43 (7.70–7.46)	7.42 (7.41–7.44)	0.438
Bicarbonates (mmol/L)	18.1 (16.8–20.3)	19 (18.2–19.9)	0.512	23.3 (21.6–25.2)	24.2 (23.2–24.7)	0.604
Base excess (mmol/L)	−6.25 (−8.15–−4.63)	−5.9 (−6.9–−4.6)	0.670	0.5 (1.9–1.35)	0.5 (−0.4–1.25)	0.514
Sodium (mmol/L)	138 (137–139.2)	139 (137.6–140)	0.729	140.6 (140–141)	140 (139.9–141)	0.828
Potassium (mmol/L)	4.9 (4.6–5.2)	4.9 (4.7–5.3)	0.827	3.7 (3.6–3.8)	3.7 (3.6–3.8)	1.000
Lactate (mmol/L)	0.9 (0.8–1.2)	1.2 (1.1–1.4)	0.012	0.8 (0.2–1)	1.1 (0.9–1.2)	0.090

**Table 3:** Median [95% CI] post-dialysis serum lactate levels (mmol/L) and median [95% CI] Δ post-dialysis/pre-dialysis serum lactate levels (mmol/L) and PDF.

	PDF Absent (n = 41)	PDF Present (n = 117)	*p*
Post-dialysis serum lactate levels	0.8 (0.7–1.0)	1.1 (0.9–1.2)	0.090
Δ Post-dialysis/pre-dialysis serum lactate	−0.1 (−0.4–0.08)	−0.2 (−0.4–−0.1)	0.490

**Table 4:** Distribution of patients according to post-dialysis serum lactate levels and PDF.

Post-Dialysis Serum Lactate Levels	PDF Absent (n = 41)	PDF Present (n = 117)	*p*
0.5–2.0 mmol/L	41	110	0.191
2.1–4.0 mmol/L	0	7	
≥4.0 mmol/L	0	0	

**Table 5:** Median [min–max] post-dialysis serum lactate levels (mmol/L) and PDF characteristics.

	1	2	3	4	5	*p*
PDF intensity	1.1 (0.8–1.9)	1.3 (0.2–2.8)	1.1 (0–3)	1.1 (0.03–2.1)	0.9 (0.2–2.2)	0.516
PDF duration	1.4 (0.2–2.8)	0.8 (0.03–2.2)	1 (0.2–2)	0.8 (0–3)	0.9 (0.3–2.7)	0.057
PDF frequency	1.3 (0.4–2.8)	0.9 (0.2–2)	1.7 (0.6–2.1)	0.7 (0.3–1.5)	0.8 (0–3)	<0.001

**Table 6:** Median [min–max] Δ post-dialysis/pre-dialysis serum lactate levels (mmol/L) and PDF characteristics.

	1	2	3	4	5	*p*
PDF intensity	0.1 (−0.1–0.3)	−0.1 (−1.5–0.6)	−0.3 (−1.8–1.2)	−0.2 (−2.2–0.9)	−0.4 (−1.9–1.1)	0.235
PDF duration	−0.05 (−1.8–0.8)	−0.3 (−2.2–1.1)	−0.1 (−1.5–0.6)	−0.3 (−1.8–1)	−0.3 (−1.8–1.2)	0.598
PDF frequency	−0.1 (−1.8–0.6)	−0.3 (−1.3–0.4)	−0.1 (−2.2–0.6)	−0.2 (−1.7–0.3)	−0.3 (−2–1.2)	0.986

**Table 7:** Median [95% CI] post-dialysis serum lactate levels (mmol/L) and median [95% CI] Δ post-dialysis/pre-dialysis serum lactate levels (mmol/L) and TIRD.

	TIRD ≤ 12 h (n = 70)	TIRD > 12 h (n = 47)	
Post-dialysis serum lactate levels	1.1 (0.8–1.2)	1.1 (0.7–1.3)	0.665
Δ Post-dialysis/pre-dialysis serum lactate	−0.2 (−0.3–0.1)	−0.3 (−0.4–0.03)	0.895

**Table 8:** Distribution of patients according to post-dialysis serum lactate levels and TIRD.

Post-Dialysis Serum Lactate Levels	TIRD ≤ 12 h (n = 70)	TIRD > 12 h (n = 47)	*p*
0.5–2.0 mmol/L	67	43	0.436
2.1–4.0 mmol/L	3	4	
≥4.0 mmol/L	0	0	

## Data Availability

The data presented in this study are available on request from the corresponding author.
